# Transoesophageal Ultrasound Assessment of Lung Aeration in Patients With Acute Respiratory Distress Syndrome

**DOI:** 10.3389/fphys.2021.716949

**Published:** 2021-09-09

**Authors:** Clément Brault, Yoann Zerbib, Loay Kontar, Julien Maizel, Michel Slama

**Affiliations:** Intensive Care Department, CHU Amiens-Picardie, Amiens, France

**Keywords:** lung ultrasound, acute respiratory distress syndrome, positive end-expiratory pressure, lung recruitment, alveolar recruitment

## Abstract

**Introduction:** The effect of positive end-expiratory pressure (PEEP) depends closely on the potential for lung recruitment. Bedside assessment of lung recruitability is crucial for personalized lung-protective mechanical ventilation in acute respiratory distress syndrome (ARDS) patients.

**Methods:** We developed a transoesophageal lung ultrasound (TE-LUS) method in which a quantitative (computer-assisted) grayscale determination served as a guide to PEEP-induced lung recruitment. The method is based on the following hypothesis: when the PEEP increases, inflation of the recruited alveoli leads to significant changes in the air/water ratio. Normally ventilated areas are hypoechoic because the ultrasound waves are weakly reflected while poorly aerated areas or non-aerated areas are hyperechoic. We calculated the TE-LUS re-aeration score (RAS) as the ratio of the mean gray scale level at low PEEP to that value at high PEEP for the lower and upper lobes. A RAS > 1 indicated an increase in ventilated area. We used this new method to detect changes in ventilation in patients with a low (<0.5) vs. high (≥0.5) recruitment-to-inflation (R/I) ratio (i.e., the ratio between the recruited lung compliance and the respiratory system compliance at low PEEP).

**Results:** We included 30 patients with moderate-to-severe ARDS. In patients with a high R/I ratio, the TE-LUS RAS was significantly higher in the lower lobes than in the upper lobes (1.20 [1.12–1.63] vs. 1.05 [0.89–1.38]; *p* = 0.05). Likewise, the TE-LUS RAS in the lower lobes was significantly higher in the high R/I group than in the low R/I group (1.20 [1.12–1.63] vs. 1.07 [1.00–1.20]; *p* = 0.04).

**Conclusion:** The increase in PEEP induces a substantial gain in the ventilation detected by TE-LUS of poorly or non-aerated lower lobes (dependent lung regions), especially in patients with a high R/I ratio.

## Introduction

Acute respiratory distress syndrome (ARDS) is characterized by a nonhomogeneous distribution of ventilation. The non-aerated lung comprises a recruitable volume (which can be re-aerated by applying the appropriate pressure) and a consolidated volume (Gattinoni et al., 2006). Furthermore, the amount of lung parenchyma that can respond to high positive end-expiratory pressure (PEEP) and/or lung recruitment maneuvers also varies widely from one ARDS patient to another. In patients with a high recruitable lung volume, the aerated lung volume will increase and the cyclical closing-reopening of the alveoli (atelectrauma) will decrease. Conversely, the application of a high level of pressure to patients with a low recruitable lung volume can lead to lung overdistention (volotrauma) and cardiac dysfunction (Gattinoni et al., 2020). Consequently, bedside assessment of lung recruitability is crucial for personalized lung-protective mechanical ventilation.

The most frequently evaluated method to assess lung recruitability is based on a computed tomography (CT) scan performed at two pressure levels. Lung recruitability is then calculated as the difference in aerated lung volume between the two scans (Gattinoni et al., 2006; Constantin et al., 2010). Although this CT method is reliable, it is not feasible in routine practice: the patient has to be moved and is exposed to ionizing radiation (Sahetya et al., 2017). A second approach is based on the analysis of multiple pressure-volume (PV) curves, starting at different end-expiratory lung volumes (EELVs) and pressures. Again, this tool is accurate but is also complicated to implement (Hess, 2015; Chen et al., 2020). More recently, Chen et al. (2020) validated a bedside method for calculating the recruitment-to-inflation (R/I) ratio and thus provide a reliable and non-invasive means of evaluating lung recruitability.

In the last decade, transthoracic lung ultrasound (TT-LUS) has been evaluated in many critical care situations, such as the diagnosis of ventilator-associated pneumonia or weaning from mechanical ventilation (Mojoli et al., 2019). In patients with ARDS, the measurement of changes in ultrasound patterns in 12 different thoracic areas and at different PEEPs provides a semiquantitative lung aeration score (from 0 to 36) that is well correlated with the CT-based lung recruitability volume (Bouhemad et al., 2011). We have developed a new method based on a similar principle, i.e., the measurement of PEEP-related changes during a transoesophageal ultrasound assessment of the lung (TE-LUS). Here, we conducted an exploratory study to assess the effects of an increase in PEEP on the lung re-aeration detected by transoesophageal lung ultrasound (TE-LUS) according to lung recruitability (assessed by the R/I ratio).

## Materials and Methods

Study population: the study was conducted in the intensive care medicine department of Amiens University Hospital (Amiens, France) from January to November 2020. All patients presenting ARDS with an arterial oxygen partial pressure to inspired oxygen fraction (P_a_O_2_/F_i_O_2_) ratio of less than 150 mmHg at a PEEP of 5 cmH_2_O. The exclusion criteria were age under 18, pregnancy, hemodynamic instability (defined as a change in vasoactive drug administration in the previous 6 h), pleural effusion, and contraindications to TE-LUS (such as oesophageal stenosis, oesophageal tumors, oesophageal varices, or gastrointestinal hemorrhage). The study was approved by the local independent ethics committee (CPP Nord-Ouest II, Amiens, CEERNI 110). In accordance with French legislation, written informed consent was obtained from the patient. Patients who were unable to provide consent prior to randomization due to orotracheal intubation or other medical conditions were informed as soon as conditions permitted.

Study procedures: all patients were ventilated in volume-control mode using V500 (Drager, Lübeck, Germany) or Servo i (Maquet, Solna, Sweden) systems. Patients received continuous intravenous sedation, analgesia, and curarization. The patients were in a semi-recumbent position, with the torso at an angle of 30–45 degrees to the horizontal. The tidal volume was set to 6 mL per kilogram of predicted body weight and the pressure plateau was kept below 28–30 cmH_2_O. The F_i_O_2_ level was adjusted to achieve arterial oxygen saturation (S_p_O_2_) of 88–92%. We evaluated airway closure during low-flow (5 L/min) insufflation, starting at a PEEP of 5 cmH_2_O. Airway closure was defined as the presence of an inflection point on the time-pressure curve, and the airway opening pressure (AOP) was defined as the pressure at this inflection point. An AOP above 5 cmH_2_O was considered to be clinically significant. We calculated the R/I ratio by applying the method recently described by Chen et al. (2020). Briefly, we abruptly decreased the PEEP (from 15 cmH_2_O or the AOP+10 cmH_2_O to 5 cmH_2_O or the AOP), in order to measure the induced change in end-expiratory lung volume (ΔEELV). We then calculated the change in lung volume (ΔV_rec_) as the difference between the measured ΔEELV and the predicted ΔEELV (i.e., the compliance at low PEEP multiplied by the change in PEEP). Next, we calculated the recruited lung’s compliance (C_rec_) as the ratio between ΔV_rec_ and the effective change in pressure (i.e., 10 cmH_2_O). Lastly, we calculated the R/I ratio as the ratio between Crec and compliance at a low PEEP. Thus, the R/I ratio quantifies the risk-benefit ratio of the application of PEEP. The higher the R/I ratio (≥ 0.5), the more the recruited lung is compliant (i.e., recruited volume is higher than alveolar overdistention). Conversely, the lower the R/I ratio (<0.5), the greater the risk of alveolar overdistention (without benefit in terms of recruitment induced by the increase of PEEP).

**FIGURE 1:**
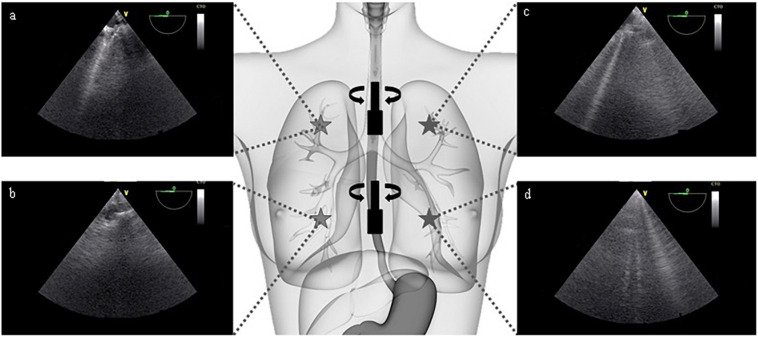
Selected transoesophageal views of the upper right **(a)**, lower right **(b)**, upper left **(c)**, and lower left **(d)** lobes of the lung (patient #10). The transducer is introduced into the esophagus to a depth of 20–25 cm and then 30–40 cm below the incisors. At each step, the transducer is turned to the left and to the right, in order to avoid the heart and view the lung parenchyma. It should be noted that all images were captured at a multiplane angle of 0 degree. The view depth was set to 15 cm. Each image was captured during a 5-s end-expiratory occlusion.

The TE-LUS assessment was performed with a VIVID 7 system (GE Medical Systems, Milwaukee, WI, United States). After removal of the gastric tube, we introduced the ultrasound transducer (9T Multi Plane Phased Array, 4.0-10.0 MHz, GE Medical Systems, Milwaukee, WI, United States) into the upper esophagus (20-25 cm below the incisors, corresponding to the ascending aortic short-axis view. We turned the transducer toward the left shoulder and then the right shoulder, corresponding, respectively, to the left upper and right upper lobes. The transducer was then placed in the mid-esophagus (30–40 cm from incisors), which corresponded to a four-chamber view. We again turned the transducer toward the left shoulder and then the right shoulder, corresponding, respectively, to the left lower and right lower lobes. To avoid interference from the heart, we made sure that the organ was not visible on the ultrasound system’s screen. To ensure that the frame rate did not change, we set the depth to 15 cm and the sector size to 90 degrees. Each image was captured during a 5-s end-expiratory occlusion at low PEEP (i.e., 5 cmH_2_O or the AOP) or high PEEP (i.e., 15 cmH_2_O or the AOP+10 cmH_2_O) (Figure 1). The ventilator settings were the same at the two PEEP levels, as were the ultrasound system settings (including the gain).

**FIGURE 2:**
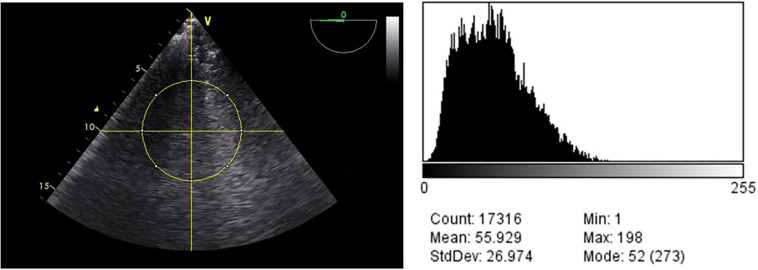
Characteristics (the mean, standard deviation, mode, and range) for the ROI, using the histogram function (patient #11).

Image analysis: in TE-LUS, normally ventilated areas are dark (i.e., hypoechoic) because the ultrasound waves are weakly reflected. Conversely, poorly aerated areas or non-aerated areas are light (i.e., hyperechoic) (Gargani and Volpicelli, 2014). We used ImageJ software (U.S. National Institutes of Health, Bethesda, MD, United States) (Schneider et al., 2012) to determine the images’ gray scale distribution. Each image was converted into a 32-bit format, corresponding to 256 bins. The region of interest (ROI) was a circle with a diameter of 150 pixels and a depth of 6 cm. The mean (SD) gray scale value at each pixel in the ROI was measured using the histogram function (Figure 2). Hence, we defined the mean gray scale value in the lower lobes and the upper lobes at a low PEEP and a high PEEP (LL_PEEPlow_, LL_PEEPhigh_, UL_PEEPlow_, and UL_PEEPhigh_). We then calculated the TE-LUS re-aeration score (RAS) as the ratio of the mean gray scale level at low PEEP to that value at high PEEP for the lower lobes (RAS_LL_) and upper lobes (RAS_UL_). A ratio higher than 1 indicates an increase in ventilated area.

Statistical analysis: Data were quoted as the median [interquartile range (IQR)]. Groups were compared using an unpaired two-sample *t*-test or the Mann-Whitney *U*-test, as appropriate. All statistical analyses were performed using GraphPad Prism (version 8.0.0, GraphPad Software, San Diego, CA, United States).

## Results

### Study Population

We included 38 patients with moderate-to-severe primary ARDS. Eight of the 38 (21%) were excluded because of pleural effusion. The main characteristics of the study population are summarized in Table 1. Twenty (67%) of the 30 patients were male, and the overall median [IQR] age was 63 [54–75]. Twenty-six (87%) patients were obese; the median body mass index was 31 [27–37] kg/m^2^. The median [IQR] P_a_O_2_/F_i_O_2_ ratio was 103 [88–128] mmHg, and 15 (50%) patients had severe ARDS. With regard to the CT scan, 27 (90%) patients had a diffuse alveolar pattern. Alveolar consolidations of the lower lobes were present in 13 (43%) patients.

**TABLE 1:** Demographic and respiratory data for the study population.

**Parameters**	**Study population (*n* = 30) Mean [IQR] or N (%)**
Demographic parameters	
Age, years	63 [54–75]
Sex, male	20 (67)
Body mass index, kg/m^2^	31 [27–37]
Obesity	26 (87%)
Cause of ARDS	
COVID-19-related ARDS	25 (83%)
Aspiration pneumonia	2 (7%)
Ventilator-associated pneumonia	2 (7%)
Unknown	1 (3%)
Respiratory parameters	
C_rs_, ml/cmH_2_O	32 [26–41]
P_a_O_2_/F_i_O_2_, mmHg	103 [88–128]
AOP > 5 cmH_2_O	11 (37)
AOP, cmH_2_O	8 [7–9]
Low PEEP, cmH_2_O	5 [5–7]
High PEEP, cmH_2_O	15 [15–17]
Computed tomography findings	
Diffuse pattern	27 (90)
Focal pattern	3 (10)
Ground-glass opacity	21 (70)
Upper lobe consolidation	4 (13)
Lower lobe consolidation	13 (43)

*AO, airway opening pressure; ARDS, acute respiratory distress syndrome; COVID-19, coronavirus disease 2019; C_rs_, respiratory system compliance; IQR, interquartile range; P_a_O_2_/F_i_O_2_, arterial oxygen partial pressure to inspired oxygen fraction ratio; PEEP, positive end-expiratory pressure.*

### Intragroup Comparisons

Eleven (37%) patients presented airway closure at a median [IQR] AOP of 8 [7–9] cmH_2_O. The median [IQR] low and high PEEPs used to calculate the R/I ratio and the TE-LUS RAS were 5 [5–7] and 15 [15–17] cmH_2_O, respectively (Table 1).

**FIGURE 3:**
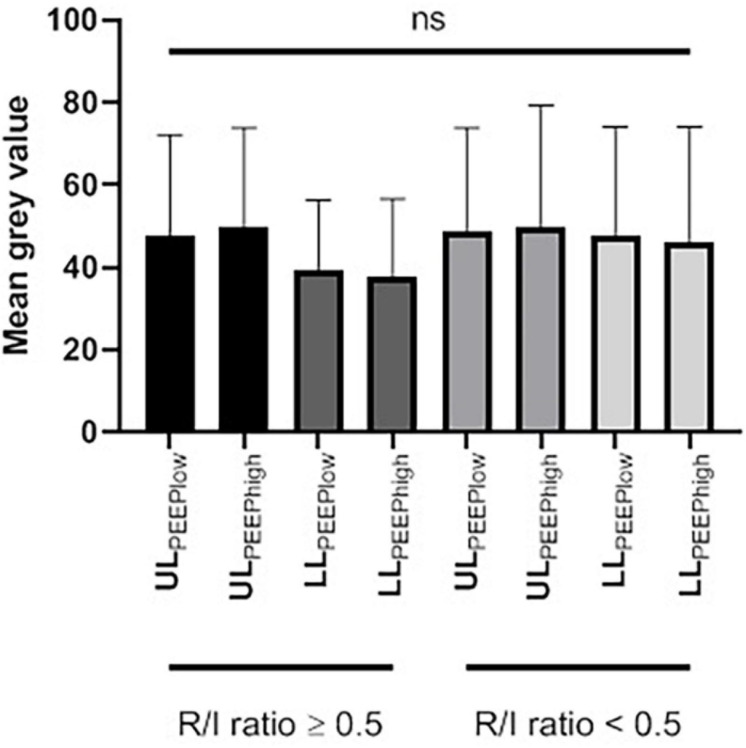
The mean gray scale value of the upper and lower lobes at a low and a high PEEP, as a function of the R/I ratio. LL, lower lobe; PEEP, positive end-expiratory pressure; ns, not significant; R/I, recruitment-to-inflation; UL, upper lobe.

**FIGURE 4:**
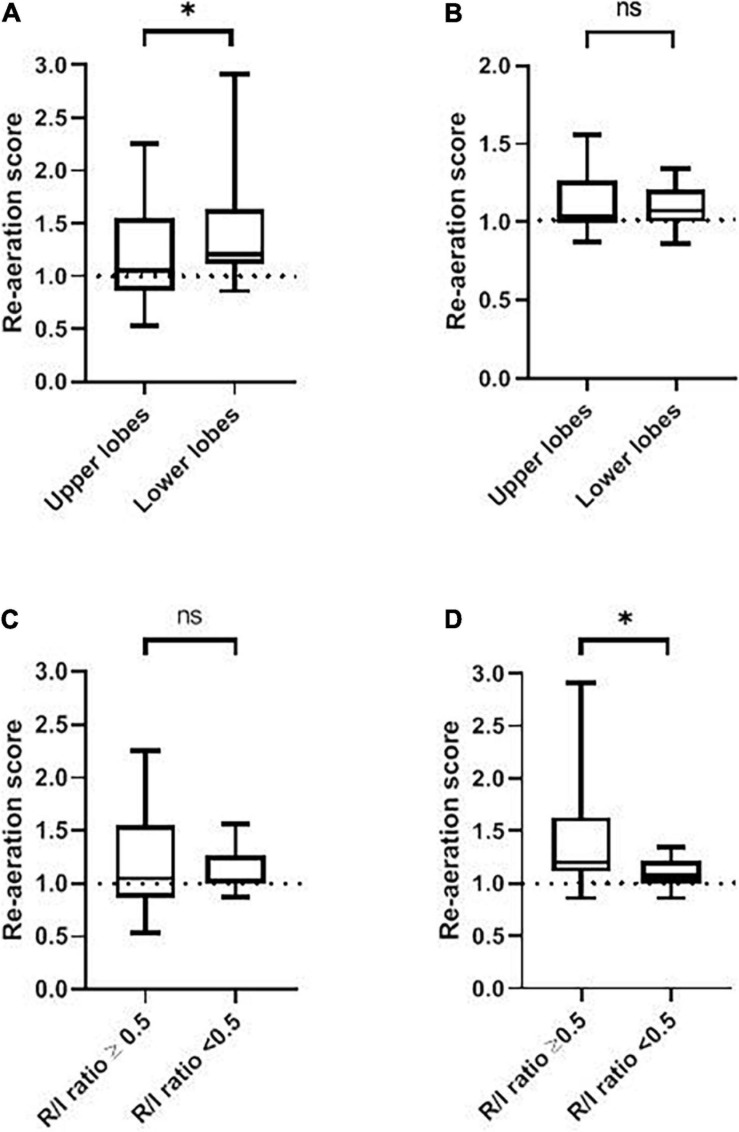
The RAS as a function of the recruitment-to-inflation ratio. **(A)** The RAS for the upper and lower lobes in patients with a high R/I ratio (≥0.5). **(B)** The RAS for the upper and lower lobes in patients with a low R/I ratio (<0.5). **(C)** The RAS for a high vs. low R/I ratio in the upper lobes. **(D)** The RAS for a high vs. low R/I ratio in the lower lobes. ns, not significant; R/I, recruitment-to-inflation. ^∗^*p* < 0.05.

Nineteen (63%) patients had a high R/I ratio (≥0.5) with a median [IQR] of 0.75 [0.58–0.83], corresponding to a high degree of recruitability. Within this group, there was no significant difference between the gray scale values for LL_PEEPlow_ vs. LL_PEEPhigh_ (40 [32–44] and 38 [27–44], respectively, *p* = 0.84) or for UL_PEEPlow_ vs. UL_PEEPhigh_ (44 [30–69] and 48 [38–56], respectively, *p* = 0.79) (Table 2 and Figure 3). The median [IQR] RAS_LL_ and RAS_UL_ were 1.20 [1.12–1.63] and 1.05 [0.89–1.38], respectively. The increase in PEEP induced a greater rise in ventilation in the lower lobes than in the upper lobes (*p* = 0.05) (Table 2 and Figure 4).

**TABLE 2:** Relationships between the R/I ratio, the mean gray scale value, and the TE-LUS RAS.

**Parameters**	**Overall study population (*n* = 30)**	**R/I ratio ≥ 0.5 (*n* = 19)**	**R/I ratio < 0.5 (*n* = 11)**	***P*-value**
R/I ratio	0.55 [0.44–0.78]	0.75 [0.58–0.83]	0.41 [0.30–0.44]	<0.001
**Mean gray scale value of LL**				
LL_PEEPlow_	39 [29–50]	40 [32–44]	39 [28–63]	0.41
LL_PEEPhigh_	39 [31–53]	38 [27–44]	42 [36–57]	0.19
LL_PEEPlow_ vs. LL_PEEPhigh_ (*p*-value)		0.84	0.82	
**Mean gray scale value of UL**				
UL_PEEPlow_	42 [30–71]	44 [30–69]	39 [31–74]	0.99
UL_PEEPhigh_	48 [35–57]	48 [38–56]	50 [39–55]	0.97
UL_PEEPlow_ vs. UL_PEEPhigh_ (*p*-value)		0.79	0.87	
**TE-LUS RAS**				
RAS_LL_	0.84 [0.76–0.94]	1.20 [1.12–1.63]	1.07 [1.00–1.20]	0.04
RAS_UL_	0.96 [0.80–1.08]	1.05 [0.89–1.38]	1.03 [0.99–1.17]	0.86
RAS_LL_ *vs.* RAS_UL_ (*p*-value)		0.05	> 0.99	

*LL, lower lobe; PEEP, positive end-expiratory pressure; R/I, recruitment-to-inflation ratio; RAS_LL_, lower lobe re-aeration score; RAS_UL_, upper lobe re-aeration score; TE-LUS, transoesophageal lung ultrasound; UL, upper lobe.*

Eleven (37%) patients had a low R/I ratio (<0.5) with a median [IQR] of 0.41 [0.30–0.44], corresponding to a low degree of recruitability. Within this group, there was no significant difference between the gray scale values for LL_PEEPlow_ vs. LL_PEEPhigh_ (39 [28-63] and 42 [36-57], respectively, *p* = 0.82) and between the gray scale value of UL_PEEPlow_ and UL_PEEPhigh_ (39 [31–74] and 50 [39–55], respectively, *p* = 0.87) (Table 2 and Figure 3). The median [IQR] RAS_LL_ and RAS_UL_ were 1.07 [1.00–1.20] and 1.03 [0.99–1.17], respectively. There was no significant difference in the increase in ventilation for the lower vs. upper lobes (*p* > 0.99) (Table 2 and Figure 4).

### Intergroup Comparisons

There were no significant differences in the gray scale values at LL_PEEPlow_, LL_PEEPhigh_, UL_PEEPlow_ and UL_PEEPhigh_ between patients with a high R/I and those with a low R/I (*p* = 0.41, *p* = 0.19, *p* = 0.99 and *p* = 0.97; respectively) (Table 2 and Figure 3).

**FIGURE 5:**
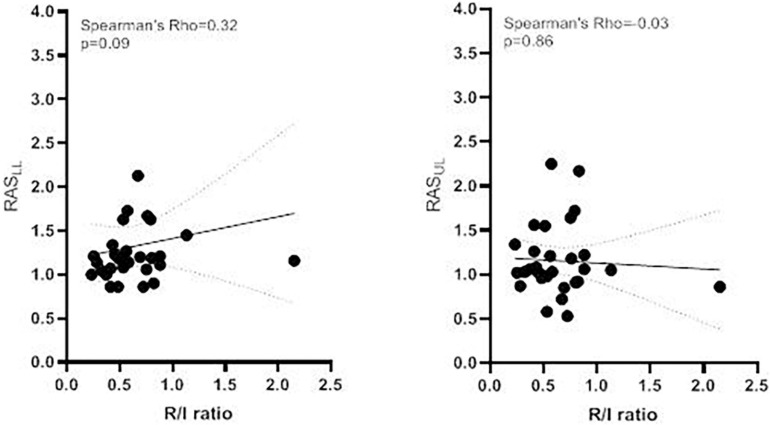
Correlation between the R/I ratio and the RAS Correlation between the R/I ratio and **(A)** the RAS_LL_ and the **(B)** RAS_UL_. The continuous line shows the linear regression (with 95% confidence interval in dashed lines). R/I, recruitment-to-inflation; RAS_LL_, lower lobe re-aeration score; RAS_UL_, upper lobe re-aeration score.

The RAS_LL_ was significantly higher in the high R/I group than in the low R/I group (1.20 [1.12–1.63] vs. 1.07 [1.00–1.20], respectively, *p* = 0.04). This is consistent with a significantly greater increase in lower lobe ventilation in the high R/I than in the low R/I group. In contrast, there was no significant difference in the RAS_UL_ between the high R/I and low R/I groups (1.05 [0.89–1.38] *vs.* 1.03 [0.99–1.17], respectively, *p* = 0.86) (Table 2 and Figure 4). We found non-significant correlations the R/I ratio and both RAS_LL_ and RAS_UL_ (Spearman’s Rho = 0.32 and 0.03, respectively, *p* > 0.05) (see Figure 5).

## Discussion

In our population of patients with moderate-to-severe ARDS, we found that the mean gray scale value of an ROI in the lower lobes and upper lobes did not depend on the PEEP or the R/I ratio. However, in patients with a high R/I ratio, the TE-LUS RAS was significantly higher in the lower lobes than in the upper lobes. Likewise, the TE-LUS RAS in the lower lobes was significantly higher in the high R/I group than in the low R/I group. These findings show that the increase in PEEP induces a substantial gain in the ventilation of poorly or non-aerated lower lobes (dependent lung regions), especially in patients with a high R/I ratio.

The literature data show clearly that TT-LUS is a reliable means (vs. PV curves or CT scan) of assessing PEEP-induced lung recruitment at the bedside in patients with ARDS (Bouhemad et al., 2011; Rode et al., 2012; Algieri et al., 2014). Furthermore, TT-LUS has several advantages: it avoids exposure to radiation exposure, the equipment is portable, and the method is non-invasive, inexpensive, and easily repeatable. Although TT-LUS is available in nearly every intensive care unit, correct interpretation of the findings requires formal training. Bouhemad et al. (2011) developed the TT-LUS RAS as a guide to the change in aeration (which is graded between 1 and 5) induced by a PEEP increase in each of 12 regions of the lung. An TT-LUS RAS of 8 or more was associated with a PEEP-induced lung recruitment of greater than 600 mL measured by PV curves (Bouhemad et al., 2011). Other methods based on TT-LUS have been described, such as measurement of the non-aerated lung surface area and the detection of alveolar consolidation in dependent lung regions during a PEEP trial (Lichtenstein et al., 2004; Gardelli et al., 2009; Stefanidis et al., 2011; Du et al., 2015; Tusman et al., 2016). (Tsubo et al., 2001a,b, 2004) used TE-LUS to estimate the change in density in dependent regions of the left lung during a PEEP trial or in the prone position. However, these methods only provided information of the dependent lung regions which are not representative of the whole lung, especially in non-focal ARDS (Bello and Blanco, 2019).

We have developed a new TE-LUS method in which a quantitative (computer-assisted) grayscale determination serves as a guide to PEEP-induced lung recruitment. The method is based on the following hypothesis: when the PEEP increases, inflation of the recruited alveoli leads to dramatic changes in the air/water ratio, which can be detected with ultrasound (Tang et al., 2017). Hence, when the PEEP is low, the ROI is hyperechoic and the mean gray scale value increases (up to a maximum value of 255) as a massive loss of alveoli aeration leads to lung consolidation and poor ultrasound transmission. Conversely, with a high PEEP, the ROI is hypoechoic and the mean gray scale value decreases (down to a minimum value of 0) because ultrasound is not transmitted through the re-aerated (gas-filled) alveoli (Aldrich, 2007; Bouhemad et al., 2007). We calculated the TE-LUS RAS (the ratio of the mean gray scale value at a low PEEP to the value at a high PEEP); a value above 1 reflects an increase in the ventilated area. We found that an increase of PEEP induced aeration gain on the whole lung, but predominantly in lower lobes and in patients with high lung recruitability. The effect of PEEP on the regional ventilation assessed by TT-LUS, CT scan and electrical impedance tomography provided conflicting results (Puybasset et al., 1998; Bouhemad et al., 2011; Bello and Blanco, 2019). Briefly, the effect of PEEP depended on the distribution of aeration loss (i.e., focal, diffuse or patchy) (Constantin et al., 2010). In a seminal TT-LUS study, Bouhemad et al. (2011) found that in diffuse ARDS, PEEP-induced lung re-aeration predominated in all but posterior and caudal regions. While in focal ARDS, lung consolidation predominated in the lower lung regions and the application of PEEP led to a significant re-aeration of these regions (Bouhemad et al., 2011). In our patients, a diffuse alveolar pattern predominated but alveolar consolidation of the lower lobes was relatively common (13 patients, 43%) which might partly explain our results.

The TE-LUS is a simple bedside tool that does not require interpretation of LUS pictures, especially when the propagation of ultrasound from the skin to the edge of the lung is hampered by adipose tissue or subcutaneous emphysema. However, several limitations of this pilot study need to be acknowledged and could partially explain the lack of correlation between the RE-LUS RAS and the R/I ratio. First, a R/I threshold of 0.5 was chosen to distinguish low and high recruitability while the median of our population was 0.55. However, the main results of the study remained unchanged using a threshold of 0.55 (see Supplementary Table 1). Second, the technique lacks spatial resolution, and ultrasound cannot penetrate deep into tissues (Gattinoni et al., 2017). This shortcoming can be exacerbated by lung oedema, pleural effusion or atelectasis in the ROI; the resulting noise interferes with the determination of the mean gray scale value. We tried to correct for these limitations (at least in part) by excluding patients with pleural effusion and by assessed only two PEEP levels (low to high) that differed by 10 cmH_2_O; this change was not perhaps large enough to elicit significant differences in the mean gray scale value. Third, our TE-LUS method inherently focused on the PEEP-induced change in aeration in the base and apex of the lung but not in the ventral and dorsal regions. Nonetheless, gravitational forces induce a ventral-to-dorsal gradient in the transpulmonary pressure; collapse predominates in the dorsal (gravity-dependent) regions, while over-inflation predominates in the ventral (gravity-independent) zones (Gattinoni and Pesenti, 2005). Fourth, when the TE-LUS RAS indicates a PEEP-induced increase in the ventilated area, it is not possible to differentiate between the further inflation of open alveoli and the reopening of previously closed alveoli (i.e., lung recruitment). Indeed, it has been reported out that LUS cannot detect lung over-inflation (Bouhemad et al., 2007; Pesenti et al., 2016; Chiumello et al., 2018). In this regard, we did not assess the effect of PEEP increase and lung re-aeration on ventilatory mechanics and gas exchange. Finally, we did not compare the TE-LUS RAS with the gold standard method for testing lung recruitability (i.e., chest CT-scan).

## Conclusion

In patients with a recruitable lung volume (according to the R/I ratio), PEEP-induced re-expansion varies from one region of the lung to another. A TE-LUS assessment with calculation of the RAS evidenced a preferential increase in ventilation in the lower lobes in patients with an R/I ratio ≥ 0.5. However, this TE-LUS technique cannot differentiate the further PEEP-induced inflation of open alveoli and the reopening of previously closed alveoli.

## Data Availability Statement

The raw data supporting the conclusions of this article will be made available by the authors, without undue reservation.

## Ethics Statement

The studies involving human participants were reviewed and approved by the CPP Nord-Ouest II, Amiens, CEERNI 110. The patients/participants provided their written informed consent to participate in this study.

## Author Contributions

CB and YZ collected the data. CB performed the analysis. CB, YZ, JM, and MS wrote the manuscript. LK, JM, and MS critically reviewed the manuscript. All authors approved the final version of the manuscript.

## Conflict of Interest

The authors declare that the research was conducted in the absence of any commercial or financial relationships that could be construed as a potential conflict of interest.

## Publisher’s Note

All claims expressed in this article are solely those of the authors and do not necessarily represent those of their affiliated organizations, or those of the publisher, the editors and the reviewers. Any product that may be evaluated in this article, or claim that may be made by its manufacturer, is not guaranteed or endorsed by the publisher.

## References

[B1] Aldrich J. E. (2007). Basic physics of ultrasound imaging. *Crit. Care Med.* 35(5 Suppl.) S131–S137. 17446771 10.1097/01.CCM.0000260624.99430.22

[B2] Algieri I. Mongodi S. Chiumello D. Mojoli F. Cressoni M. Via G. (2014). CT scan and ultrasound comparative assessment of PEEP-induced lung aeration changes in ARDS. *Crit. Care* 18(Suppl. 1):285.

[B3] Bello G. Blanco P. (2019). Lung ultrasonography for assessing lung aeration in acute respiratory distress syndrome: a narrative review. *J. Ultrasound Med.* 38 27–37. 10.1002/jum.14671 29732586

[B4] Bouhemad B. Brisson H. Le-Guen M. Arbelot C. Lu Q. Rouby J.-J. (2011). Bedside ultrasound assessment of positive end-expiratory pressure-induced lung recruitment. *Am. J. Respir. Crit. Care Med.* 183 341–347. 10.1164/rccm.201003-0369oc 20851923

[B5] Bouhemad B. Zhang M. Lu Q. Rouby J.-J. (2007). Clinical review: bedside lung ultrasound in critical care practice. *Crit. Care* 11:205. 17316468 10.1186/cc5668 PMC2151891

[B6] Chen L. Del Sorbo L. Grieco D. L. Junhasavasdikul D. Rittayamai N. Soliman I. (2020). Potential for lung recruitment estimated by the recruitment-to-inflation ratio in acute respiratory distress syndrome. a clinical trial. *Am. J. Respir. Crit. Care Med.* 201 178–187. 10.1164/rccm.201902-0334oc 31577153

[B7] Chiumello D. Mongodi S. Algieri I. Vergani G. L. Orlando A. Via G. (2018). Assessment of lung aeration and recruitment by CT scan and ultrasound in acute respiratory distress syndrome patients. *Crit. Care Med.* 46 1761–1768. 10.1097/ccm.0000000000003340 30048331

[B8] Constantin J.-M. Grasso S. Chanques G. Aufort S. Futier E. Sebbane M. (2010). Lung morphology predicts response to recruitment maneuver in patients with acute respiratory distress syndrome. *Crit. Care Med.* 38 1108–1117. 10.1097/ccm.0b013e3181d451ec 20154600

[B9] Du J. Tan J. Yu K. Wang R. (2015). Lung recruitment maneuvers using direct ultrasound guidance: a case study. *Respir. Care* 60 e93–e96. 25406343 10.4187/respcare.03056

[B10] Gardelli G. Feletti F. Gamberini E. Bonarelli S. Nanni A. Mughetti M. (2009). Using sonography to assess lung recruitment in patients with acute respiratory distress syndrome. *Emerg. Radiol.* 16 219–221. 10.1007/s10140-008-0734-1 18830644

[B11] Gargani L. Volpicelli G. (2014). How i do it: lung ultrasound. *Cardiovasc. Ultrasound* 12 25. 24993976 10.1186/1476-7120-12-25 PMC4098927

[B12] Gattinoni L. Pesenti A. (2005). The concept of “baby lung”. *Intensive Care Med.* 31 776–784. 10.1007/s00134-005-2627-z 15812622

[B13] Gattinoni L. Caironi P. Cressoni M. Chiumello D. Ranieri V. M. Quintel M. (2006). Lung recruitment in patients with the acute respiratory distress syndrome. *N. Engl. J. Med.* 354 1775–1786. 16641394 10.1056/NEJMoa052052

[B14] Gattinoni L. Collino F. Maiolo G. Rapetti F. Romitti F. Tonetti T. (2017). Positive end-expiratory pressure: how to set it at the individual level. *Ann. Transl. Med.* 5:288. 10.21037/atm.2017.06.64 28828363 PMC5537121

[B15] Gattinoni L. Marini J. J. Quintel M. (2020). Recruiting the acutely injured lung: how and why? *Am. J. Respir. Crit. Care Med.* 201 130–132. 10.1164/rccm.201910-2005ed 31661307 PMC6961753

[B16] Hess D. R. (2015). Recruitment maneuvers and PEEP titration. *Respir. Care* 60 1688–1704. 10.4187/respcare.04409 26493593

[B17] Lichtenstein D. A. Lascols N. Mezière G. Gepner A. (2004). Ultrasound diagnosis of alveolar consolidation in the critically ill. *Intensive Care Med.* 30 276–281. 10.1007/s00134-003-2075-6 14722643

[B18] Mojoli F. Bouhemad B. Mongodi S. Lichtenstein D. (2019). Lung ultrasound for critically ill patients. *Am. J. Respir. Crit. Care Med.* 199 701–714. 30372119 10.1164/rccm.201802-0236CI

[B19] Pesenti A. Musch G. Lichtenstein D. Mojoli F. Amato M. B. P. Cinnella G. (2016). Imaging in acute respiratory distress syndrome. *Intensive Care Med.* 42 686–698. 27033882 10.1007/s00134-016-4328-1

[B20] Puybasset L. Cluzel P. Chao N. Slutsky A. S. Coriat P. Rouby J.-J. A. (1998). Computed tomography scan assessment of regional lung volume in acute lung injury. *Am. J. Respir. Crit. Care Med.* 158 1644–1655. 10.1164/ajrccm.158.5.9802003 9817720

[B21] Rode B. Vučić M. Siranović M. Horvat A. Krolo H. Kelečić M. (2012). Positive end-expiratory pressure lung recruitment: comparison between lower inflection point and ultrasound assessment. *Wien. Klin. Wochenschr.* 124 842–847. 10.1007/s00508-012-0303-1 23229578

[B22] Sahetya S. K. Goligher E. C. Brower R. G. (2017). Fifty years of research in ARDS. Setting positive end-expiratory pressure in acute respiratory distress syndrome. *Am. J. Respir. Crit. Care Med.* 195 1429–1438. 28146639 10.1164/rccm.201610-2035CI PMC5470753

[B23] Schneider C. A. Rasband W. S. Eliceiri K. W. (2012). NIH image to imagej: 25 years of image analysis. *Nat. Methods* 9 671–675. 10.1038/nmeth.2089 22930834 PMC5554542

[B24] Stefanidis K. Dimopoulos S. Tripodaki E.-S. Vitzilaios K. Politis P. Piperopoulos P. (2011). Lung sonography and recruitment in patients with early acute respiratory distress syndrome: a pilot study. *Crit. Care* 15:R185. 21816054 10.1186/cc10338 PMC3387628

[B25] Tang K.-Q. Yang S.-L. Zhang B. Liu H.-X. Ye D.-Y. Zhang H.-Z. (2017). Ultrasonic monitoring in the assessment of pulmonary recruitment and the best positive end-expiratory pressure. *Medicine* 96:e8168. 10.1097/md.0000000000008168 28953669 PMC5626312

[B26] Tsubo T. Sakai I. Suzuki A. Okawa H. Ishihara H. Matsuki A. (2001a). Density detection in dependent left lung region using transesophageal echocardiography. *Anesthesiology* 94 793–798. 10.1097/00000542-200105000-00017 11388530

[B27] Tsubo T. Yatsu Y. Suzuki A. Iwakawa T. Okawa H. Ishihara H. (2001b). Daily changes of the area of density in the dependent lung region – evaluation using transesophageal echocardiography. *Intensive Care Med.* 27 1881–1886. 10.1007/s00134-001-1115-3 11797023

[B28] Tsubo T. Yatsu Y. Tanabe T. Okawa H. Ishihara H. Matsuki A. (2004). Evaluation of density area in dorsal lung region during prone position using transesophageal echocardiography. *Crit. Care Med.* 32 83–87. 10.1097/01.ccm.0000104944.18636.b2 14707563

[B29] Tusman G. Acosta C. M. Costantini M. (2016). Ultrasonography for the assessment of lung recruitment maneuvers. *Crit. Ultrasound J.* 8:8. 27496127 10.1186/s13089-016-0045-9 PMC4975737

