# Supplementary material for: Transoesophageal Ultrasound Assessment of Lung Aeration in Patients With Acute Respiratory Distress Syndrome
Source: Front Physiol. 2021 Sep 9;12:716949. doi: 10.3389/fphys.2021.716949 (PMC8458769; doi:10.3389/fphys.2021.716949)
Supplement: Supplementary file 1 [file Table_1.DOCX]

**Supplementary Table 1:**

TE-LUS RAS in patients with a low or high lung recruitability when the threshold of R/I ratio was 0.55.

| **Parameters** | **R/I ratio ≥0.55**  **(n=16)** | **R/I ratio <0.55**  **(n=14)** | ***P* value** |
| --- | --- | --- | --- |
| R/I ratio | 0.78 [0.69-0.84] | 0.42 [0.33-0.49] | **<0.001** |
| TE-LUS RAS |  |  |  |
| RAS_LL_ | 1.20 [1.13-1.64] | 1.09 [1.00-1.21] | **0.05** |
| RAS_UL_ | 1.05 [0.90-1.32] | 1.03 [0.98-1.21] | 0.44 |
| RAS_LL_ *vs.* RAS_UL_ (*p* value) | 0.09 | 0.68 |  |

R/I: recruitment-to-inflation ratio; RAS_LL_: lower lobe re-aeration score; RAS_UL_: upper lobe re-aeration score; TE-LUS: transesophageal lung ultrasound.
